# Characteristics and Potentiality of Human Adipose-Derived Stem Cells (hASCs) Obtained from Enzymatic Digestion of Fat Graft

**DOI:** 10.3390/cells8030282

**Published:** 2019-03-25

**Authors:** Pietro Gentile, Maria Serena Piccinno, Claudio Calabrese

**Affiliations:** 1 Department of Surgical Science, University of Rome Tor Vergata, Rome 00133, Italy; 2 Scientific and Technological Park fo Medicine “Mario Veronesi”, via 29 Maggio, 6, 41037 Mirandola, Italy; mariaserena.piccinno@tpm.bio; 3 The Oncologic and Reconstructive Surgery Breast Unit, Oncology Department, Careggi University Hospital, Firenze 50134, Italy; claudiocalabrese.it@gmail.com; 4 San Rossore Breast Unit, Pisa 56122, Italy

**Keywords:** ASCs, adipose-derived stem cells, SVFs, stromal vascular fraction cells, enzymatic digestion, personalized medicine, characteristics of adipose-derived stem cells

## Abstract

Human adipose-derived stem cells localize in the stromal-vascular portion, and can be ex vivo isolated using a combination of washing steps and enzymatic digestion. For this study, we undertook a histological evaluation of traditional fat graft compared with fat graft enriched with stromal vascular fraction cells isolated by the Celution™ system to assess the interactions between cells and adipose tissue before the breast injection. In addition, we reported on histological analyses of biopsies derived from fat grafted (traditional or enriched with SVFs) in the breast in order to assess the quality of the adipose tissue, fibrosis and vessels. The hASCs derived from enzymatic digestion were systematically characterized for growth features, phenotype and multi-potent differentiation potential. They fulfill the definition of mesenchymal stem cells, albeit with a higher neural phenotype profile. These cells also express genes that constitute the core circuitry of self-renewal such as OCT4, SOX2, NANOG and neurogenic lineage genes such as NEUROD1, PAX6 and SOX3. Such findings support the hypothesis that hASCs may have a potential usefulness in neurodegenerative conditions. These data can be helpful for the development of new therapeutic approaches in personalized medicine to assess safety and efficacy of the breast reconstruction.

## 1. Introduction

Used for different therapeutic procedures aimed at treating different forms of tissue damage, the autologous transplant of adipose tissue is indeed a solution that delivers. Recent studies have demonstrated that using Adipose-Derived Stem Cells (ASCs) can enhance tissue regeneration potentiality [1]. Subcutaneous adipose tissue has a significant edge over other Mesenchymal Stem Cells (MSCs) because it is easily accessible, while posing the least amount of discomfort to the patient and being easy to use with local anesthesia. Moreover, it is easy to isolate the target stem cells from the tissue that has been harvested [2,3]. A higher quantity of stem cells has been observed in adipose tissue compared to bone marrow [4]. MSCs are essentially cells that renew on their own, in addition to being multipotent. They have the ability to split into cells of mesenchymal origin in vitro; this includes chondrocytes, adipocytes and osteoblasts. They can also augment different tissues in vivo including fat, bone, muscle and cartilage [5]. Human Adipose-Derived Stem Cells (hASCs) are localized in Stromal Vascular Fraction (SVF) of subcutaneous adipose tissue [6], which has a heterogenous mesenchymal cell set [7,8]. When Stromal Vascular Fraction cells (SVFs) are seeded into a culture, a subset of elongated cells begins to adhere to the tissue culture plastic ware. These cells can be further purified using a combination of washing steps and culture expansion, with media similar to the ones used for bone marrow MSCs. The washing and culture expansion procedures can be used in order to deplete most of the hematopoietic cell population from the SVF cells and differentiate them into classical mesodermal tissues (like bone, fat and cartilage) after isolation and under specific stimuli [9]. Recent reports suggest that with appropriate stimuli, hASCs can differentiate into pancreatic cells, hepatocytes, neurons and cardiomyocytes [10,11]. They can further hold clinical potential in relation to osteogenesis [12], vasculogenesis [13] and other neuronal repair models [14]. The ISCT (International Society of Cell Therapy) has devised a set guideline to define MSCs based on their immunophenotype, plastic adhesion properties, and multi-potential differentiation [15,16]. In this study we reported the histological evaluation of whole/dialized-filtered subcutaneous fat graft (FAT), fat graft enriched with stromal vascular fraction cells (FAT + SVF) isolated by the Celution™ 800/CRS System (Cytori Therapeutics Inc., San Diego, CA, USA, http://www.cytoritx.com) compared with traditional fat graft (without filtration/dialization) [17] to assess the interactions between cells and adipose tissue before the breast injection. The Celution 800/CRS System is a CE-Marked medical technology developed by Cytori to automate and standardize the extraction and concentration of adult SVFs in a clinical setting [17]. The Celution System, based on the enzymatic digestion of fat tissue, enables real-time access to autologous SVFs facilitating cell therapy and fat enrichment [17]. The procedure permits the reimplantation of a patient’s own fat enriched with SVFs through a single surgical procedure [17]. In addition, we report a histological analysis of biopsies derived from fat grafted in the breast (traditional or enriched with SVFs) in order to assess the quality of the adipose tissue, fibrosis and vessels. The hASCs derived from fat enzymatic digestion have been systematically characterized for growth features, phenotype and multi-potent differentiation potential. In this study we reported that a fat graft enriched with stromal vascular fraction cells (FAT + SVF group), although structurally and cellularly comparable to original adipose tissue, presents a prevalent presence of mature vessels (α-SMA+ vessels). This phenomenon was not totally reproducible into FAT groups that have higher, but no significant, number of CD31+ vessels (immature vessels), in comparison with with α-SMA+ vessels. The resulting hASCs have cell surface markers and differentiation properties which are typical of MSCs, but are highly enriched in classical neuronal protein markers. These data can be helpful for the development of new therapeutic approaches in personalized medicine to assess the safety and efficacy of the breast reconstruction.

## 2. Materials and Methods

### 2.1. Patients

Fat tissue samples were taken at Breast Surgical Unit of the Careggi Hospital, Florence, Italy, before and after surgical injection of fat graft in patients affected by outcomes of breast reconstruction. All patients gave their informed written consent, and the samples of fat graft (traditional or enriched with SVFs) were processed in accordance with the ethical committee of the Careggi Hospital and the principles of the Declaration of Helsinki. The study revolves around the data collected from 23 females. The age range for the participants was set between 28 and 80 years. The presence of HIV 1 and 2, along with HBV, HCV and cytomegalovirus, was previously confirmed in all specimens. The tests came back negative. Before the surgical injection, fat tissue samples, harvested by liposuction, were taken from abdomen. After the surgical injection, fat tissue samples, harvested by punch biopsy, were taken from the treated breast. The Body Mass Indices (BMIs) for all patients were below 30, indicating non-obese patients.

### 2.2. Fat Grafting Preparation

The process to prepare the tissue and cell normally includes two phases. The first begins with a liposuction conducted via a 715.4 mL syringe with a 250 mL/1080 mL range. It is administered using 3 mm cannulas in the abdominal area. Aseptic technique was maintained during the process, and the plunger of the syringe (60 mL) was withdrawn and closed with the use of a cap to conceal the tip. Half of the lipoaspirate (46–234 mL average) was placed into the tissue collection container of the Celution™ 800/CRS System (Cytori Therapeutics Inc., San Diego, CA, USA). Through a wash cycle, blood and free lipid was removed from the tissue and the Celase™ 835/CRS Reagent was added to enzymatically digest the tissue which released SVFs. After additional wash and centrifugation cycles, 4–5 mL of the SVFs suspension was extracted from the system. In the second phase, the remaining part of lipoaspirate was added to the tissue collection container and a washing step was automatically carried out. Once completed, the 4–5 mL of SVFs suspension was added and mixed with the washed fat graft, resulting in approximately 429.61 mL (range 60 mL/620 mL) of SVFs-enhanced fat tissue for grafting. This newly processed cell-enhanced fat graft typically consists of 25–30% water, which will be reabsorbed by the body in the post-operative period. This overall process is controlled through automated sensors and processing algorithms that ensure standard handling of the tissue and cells, and that the process is completed within 160 min. The SVFs-enhanced fat graft was transferred into 10 mL syringes and aseptically re-injected into the patient using specific micro-cannulas for implantation. One of the main reasons why this technique of SVFs-enhanced fat graft was used by the authors is that there was less resorption of the injected fat graft compared to a non-enhanced fat graft. The resorption rate reported over the first year was highly variable, i.e., 37% when SVF-enhanced fat grafts were used and 61% for non-enhanced fat grafts [17]. To prevent resorption, it is crucial to perform each step of the procedure carefully, paying close attention to the technical details. The donor site region was infiltrated with a cold saline solution containing 1 mL of adrenaline per 500 mL of saline solution without lidocaine or carbocaine to reduce bleeding during the procedure. An inverse relationship has been observed between the blood amount in the lipoaspirate and the viable number of adipocytes [17]. Adipose tissue was removed after 5 min using a 3-mm-diameter cannula and a 60-cc Toomey syringe. The authors re-injected the SVFs-enhanced adipose tissue using specific micro-cannulas (1.5 mm in diameter) for implantation. The fat injection was performed using the “Gentle technique” based on a slow and gentle injection implanting linear deposits of fat graft in the suprafascial, retroglandular e intraglandular space [18]. For this reason, the SVF-enhanced fat graft was implanted in multiple tunnels with slow and controlled movements through different entrances (inframammary fold, supero and infero-external quadrant, supero and infero-internal quadrant, periareolar) to underline the importance of a non-traumatic procedure to maximize the integrity of the grafted fat, and to maximize the contact surface between the lipoaspirate and the host’s capillaries [17,18]. The diffusion of nutrients from neighboring capillaries is essential for adipocyte survival, and favors their integration with the surrounding tissue [17,18].

### 2.3. Cell Pellet and Lipoaspirate Paraffin-Embedding

In this study, we considered a total of 34 samples (study group) comprising 23 fat samples and 11 samples cellular suspension of SVFs (SVFs pellet). Eleven fat samples of both whole/dialized-filtered lipoaspirate (FAT) and 12 samples of SVF-enhanced dialized-filtered lipoaspirate (FAT + SVF) were obtained by either Celution™ technology application. The control group comprised 10 samples of traditional lipoaspirate (without filtration/dialization/washing/centrifugation). All fat tissue samples were taken from 23 patients. These samples were transferred at 4 °C from Careggi hospital, Florence, Italy, to the cell biology laboratories and advanced oncology therapies of University of Modena, Italy for histological and immunohistochemical analysis. Then, FAT and FAT + SVF samples were fixed in 10% buffered formalin for 2 days and embedded in paraffin, while samples containing SVFs alone were suspended in 0.2% of Bovine Serum Albumin (BSA) and centrifuged at 1200 rpm for 10 min. Then, cell pellets were fixed in 10% of formalin and included in paraffin.

### 2.4. Cell Pellet and Lipoaspirate Oct-Embedding

Samples of FAT, SVFs (in this case as pellet suspension) and FAT + SVF were obtained from 3 patients (included in the study group) and transferred in cryo mold, mixing in optimum cutting temperature (OCT) (Tissue-Tek^®^ O.C.T. Compound, Sakura^®^ Finetek USA Inc. Torrance, CA, USA) and flash-freezing by dry ice/ethanol bath.

### 2.5. Hystological Analyses of Fat, SVF and Fat + SVF Samples

Sections of 5-µm thickness and 10-µm paraffin of FAT, SVFs and FAT + SVF were obtained and stained with Hematoxylin and Eosin for histological analysis. For paraffin embedding sections only, anti-CD31 (mouse-anti human CD31 antibody; 1:50), CD34 (rabbit anti-human CD34 antibody; 1:100) and CD45 (mouse-anti human CD45 antibody; 1:45), single immune-stains were performed, as previously described for mammary biopsies analysis. Analyses were performed using 400× magnification. Scorings were performed by quantifying the percentage of DAB stained areas respect of nuclear areas for 400× high power field (n = 10/each specimen) using the public software ImmunoRatio (http://153.1.200.58:8080/immunoratio/).

### 2.6. Histological Analyses for Mammary Biopsies

After surgical injection, mammary biopsies were obtained from 11 patients that received transplants of FAT and 12 patients that received transplant of FAT + SVF grafts. Samples were fixed in 10% formalin and embedded in paraffin. Sections of 5-µm paraffin were stained with Hematoxylin and Eosin (H&E) for histological analysis. To detect vessels into sample tissues, immunohistochemistry using anti-CD31 antibody and anti-αSMA antibody was performed. Sections of 5 µm paraffin were dehydrated and stained with mouse-anti human CD31 (1:50) or rabbit anti human αSMA (1:200) using a goat anti–mouse or goat anti-rabbit biotinylated secondary Ab (1:200) and an avidin-biotin-horseradish peroxidase detection system. Retrievals were performed by proteinase k 1µg/mL for 5 min at room temperature and nonspecific binding were blocked using 10% new calf serum and 10% blocking reagent. The primary antibody was applied overnight in a 0.1% albumin bovine serum (BSA). Following incubation with secondary Ab and quenching solution, slides then were incubated with Vectastain ABC and color development was performed by DAB stain and Harris hematoxylin counterstain. Negative control specimens were stained with a mouse or rabbit isotypic IgG primary Ab. Stained slides were then examined by Zeiss Axioskop microscope. Photomicrographs were acquired by Axiocam IcC3 color camera and Axiovision 4.82 software visualization. Analyses were performed using 100× magnification. Scoring was performed by counting CD31+ vessels/100× high power field and counting α-SMA+ vessels/100× high power field (n = 10/each specimen). An addition CD31-novared/αSMA-brown double stain was performed to detect the mature vessels. Sirius Red staining was introduced to value the fibrotic areas inside the transplanted tissues. Sections of 5-µm paraffin were dehydrated and stained for 3 min with Carazzi Haematoxylin and then Sirius red (0.1% in picric acid) for 10 min at room temperature. After two washes in acidified water (0.5% *v*/*v* in acetic acid), slides were mounted with coverslips and observed by microscopical examination. Analyses were performed on 100× high power field (n = 10/each specimen) and the percentage of red stained was quantified areas by ImageJ software analysis.

### 2.7. RNA Extraction and qRT-PCR Analyses

Total cellular RNAs were extracted by SVF-enhanced fat graft using TRI Reagent^®^ (Sigma-Aldrich, St. Louis, MO, USA), according to the manufacturer’s instruction. RNA purity and quantity were assessed by Nanodrop (Fisher Scientific) (A260/A280 1.8-2 was considered suitable for further analysis), possible contaminating DNA was removed, and cDNA was prepared from 1 μg of RNA using High Capacity RNA-to-cDNA Kit (Applied Biosystems, Foster City, CA, USA). Quantifications of all gene transcripts were performed by real-time retro-transcriptional polymerase chain reaction (Real Time RT-PCR) using a TaqMan^®^ Array Plate 32 (Life Technologies, Paisley, UK, www.lifetechnologies.com) on “Step One Plus™ (Applied Biosystems) for the expression of 18s rRNA, GAPDH, HPRT1, GUSB detection as the internal control. The primer pairs used were: (a) SOX2, Hs01053049_s1; (b) NANOG, Hs04260366_g1; (c) OCT4, Hs04260367_gH; (d) NestinHs04187831_g1; (e) NeuroD1, Hs01922995_s1; (f) PAX6, Hs00240871_m1; (g) SOX3,Hs00271627_s1; (h)SSEA1, Hs01106466_s1; (i) Musashi1, Hs01045894_m1; (j) CD90, Hs00264235_s1 (Life Technologies). PCR conditions consisted of 1 cycle of 50 °C for 2 min, followed by exposure at 95 °C for 10 min, 40 cycles of 95 °C for 15 s, and 60 °C for 1 min. HPRT1 and GUSB were used as invariant housekeeping genes. The quantitative expression of genes of interest relative to the housekeeping gene was calculated. This reference gene, which is also known as endogenous control, provided a basis for normalizing sample-to-sample differences. The data were only used if the calculated PCR efficiency ranged between 1.85 and 2.0. Template and reverse transcription negative controls were also included in all amplification experiments.

### 2.8. Statistical Analysis

Data are expressed as mean values +/- standard error of the mean (SEM). Statistical significance was determined by a two-tailed Student t test. A p value of <0.05 was used for define the statistical significance.

## 3. Results

### 3.1. Histological Analysis of Fat Graft (±SVF) before Transplantation

**Figure 1:**
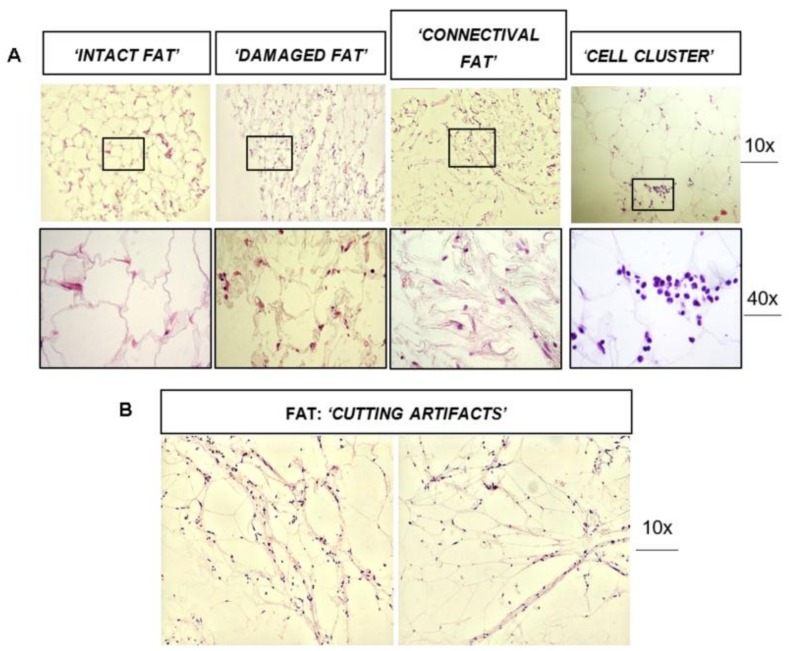
Histological analysis of fat graft (±SVF) before transplantation performed by H&E staining. (**A**) The ‘intact fat’ (normal-shaped adipocytes), the ‘damaged fat’ (irregular-shape adipocytes, with irregular cytoplasmic rims), the ‘connectival fat’ (stromal scaffolding of adipose tissue), ‘cell clusters’ (small group >15 cells of round shaped cells within the fat context) in comparison. (**B**) Fat with relevant damaged and then artifacts.

H&E staining was performed for FAT, SVFs pellets and FAT + SVFs samples (n = 34). The following parameters were assessed for FAT and FAT + SVF samples: (1) the percentage (%) of ‘intact fat’; (2) the % of ‘damaged fat’; (3) the presence of connective associated fat tissue (‘connectival fat’); (4) the presence of fat associated ‘cell clusters’ for FAT + SVFs samples. Figure 1A shows representative images of ‘intact fat’, ‘damaged fat’, ‘connective associated fat tissue’ and ‘fat associated cell cluster’.

**Figure 2:**
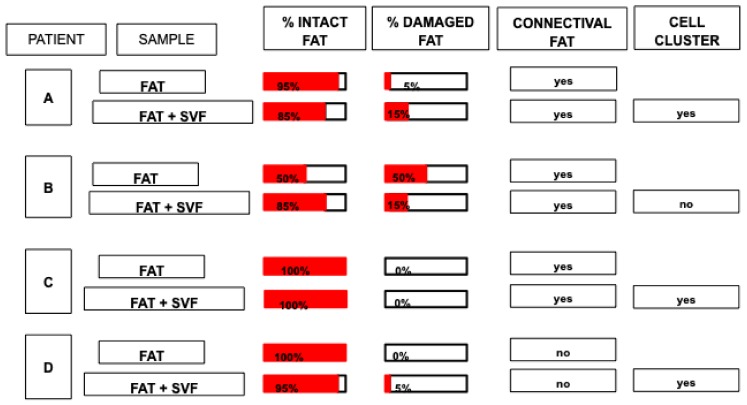
A summary of a histological analyses of randomized selection of FAT and FAT + SVF samples conventionally defined by the letter A, B, C, D (n = 4). All samples were composed predominantly by ‘intact fat’ and 3 out of 4 samples displayed a variable fraction of ‘damaged fat’.

The ‘intact fat’ represents the part of lipoaspirate composed by normal-shaped adipocytes, versus the ‘damaged fat’ composed of irregular-shape adipocytes with irregular cytoplasmic rims. The ‘connective associated fat tissue’ represents the stromal scaffolding of adipose tissue, while fat associated ‘cell clusters‘ identify small group (>15 cells) of round shaped cells within the fat context. From the 5 samples originally included, one was excluded from study because of the relevant damaged and then artifacts. Figure 2 reports a summary of a histological analyses of randomized selection of FAT and FAT + SVFs samples conventionally defined by the letter A, B, C, D (n = 4). All samples were composed predominantly by ‘intact fat’, and 3 out of 4 samples displayed a variable fraction of ‘damaged fat’. In particular, samples from patient B resulted with the highest damage mostly in the FAT alone specimens. The other samples revealed low levels (<15%) of damaged fat, suggesting that analyzed samples were histologically well preserved. This is also true comparing adipose tissues with or without SVF supplementation, indicating this latter step before surgical implementation does not harm fat graft. We then focused on fat associated connective tissue; in all collected specimens (except D), we could detect the presence of connective tissue, which may be derived from the action of lipoaspiration. This may be also linked to the anatomical site from where the fat has been harvested and, since this study is done in a blinded, way we cannot exclude that sample D was taken from a different site in comparison with A, B, C. Considering the cell clusters as parameters, we realized that those elements were exclusively present in the FAT + SVF fraction in 3 out of 4 considered specimens; this micro-anatomical aspect was absent in the FAT only specimens. Therefore, we presume that these groups of cellular elements may represent the supplemented SVFs fraction mixed with the fat tissue. Currently, we do not have an explanation for sample B, which appeared to be devoid of cell clusters. It would be interesting to combine these findings with a clinical follow up for this specific patient to assess the fat graft performance. 

### 3.2. Histological Analysis of SVFs Pellet 

**Figure 3:**
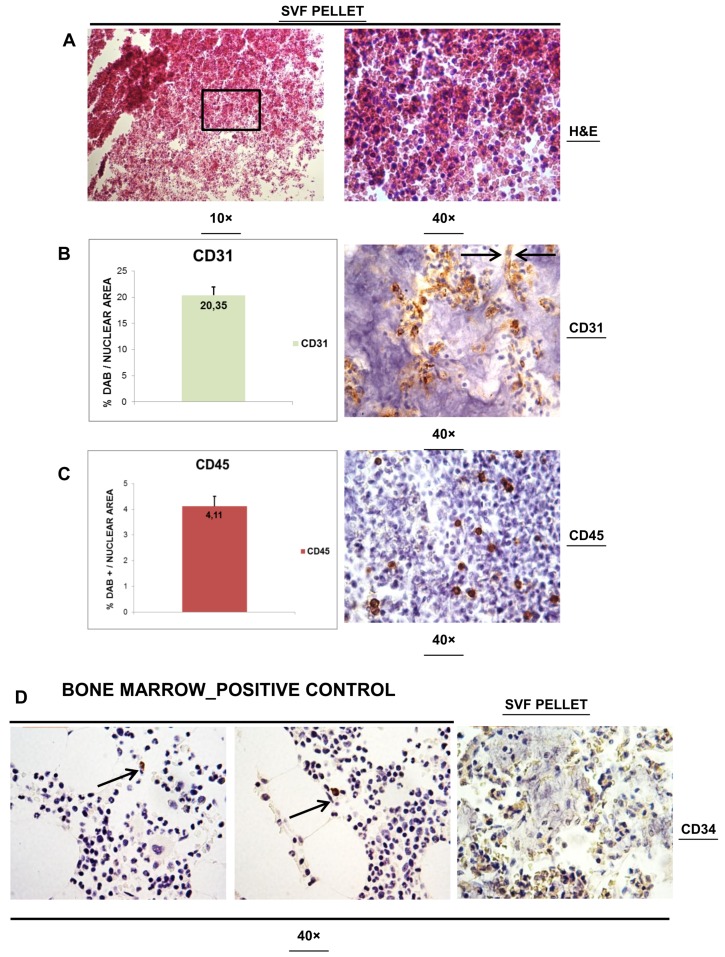
Paraffin-embedding SVFs alone as cell pellet. (**A**) The images of H&E stain for SVFs pellets. (**B**) The image (400× of magnification) of CD31 stain and its quantification. In detail, arrow indicated CD31+ cellular aggregates as tube-like distribution with flattering nuclei associated with CD31 stain of membrane. This feature resembles the aspect of transversal section of endothelium. (**C**) A representative specimen stained for CD45 (400× of magnification). (**D**) Histological stain was set-up considering human bone marrow biopsies. (Left and central panel) Marrow cellularity retained rare positivity for CD34+ small round cells (arrow). Rare positivity was instead visualized on endothelial like structure. Having set-up the staining, we than applied anti-CD34 antibody into SVF pellet (right panel).

After analysis of FAT (±SVF) samples, we evaluate paraffin-embedding SVFs alone as a cell pellet. Figure 3A displays the images of H&E stain for SVFs pellets. As expected, numerous round shaped cells were easily identifiable. Cells appeared to be homogeneous in shape and size, and were mixed with red blood cells. Nucleus and cytoplasm appeared intact, suggesting that the entire processing did alter the nucleated cell quality. Having confirmed the histological features of SVFs in pellet, we then performed three immunohistochemical stains for CD31, CD34 and CD45 as endothelial (CD31, CD34), stem cells (CD34) and leukocyte (CD45) markers, respectively. Figure 3B displays the image (400× of magnification) of CD31 stain and its quantification. All samples show two distinct features. On one side, CD31+ cells were scattered, and on the other, we could detect more evident CD31+ cellular aggregates. Several of these aggregates displayed a tube-like distribution with flattering nuclei associated with CD31 stain of membrane. This feature resembles the aspect of transversal section of endothelium (arrows). The quantification of stained areas reveals a positivity of 20.35 ± 1.62% for CD31 antigen. Figure 3C displays a representative specimen stained for CD45 (400× of magnification). As clearly shown, disperse CD45+ round shaped cells were present. The microscopical quantification of these cellular elements allowed us to define average levels of 4.11 ± 0.39%. We then evaluated the presence of CD34+ cells within SVF, which was possibly representative of hematopoietic precursors, as well as of endothelial cells. A histological stain was set-up considering human bone marrow biopsies. As seen in Figure 3D (left and central panel) marrow cellularity retained rare positivity for CD34+ small round cells (arrow). Rare positivity was instead visualized on endothelial like structure. Having set-up the staining, we than applied anti-CD34 antibody into SVFs pellet (Figure 3D, right panel). In this case, the positivity appeared more prevalent but weaker than in the marrow, and it seemed to be related to enlarged cellular elements dispersed within the matrix.

The histological analyses were mostly performed on paraffin-embedded samples versus OCT-embedded samples because of initial discouraging results. The cutting OCT-embedding samples was negatively impacting the H&E analyses, producing frequent ‘cutting artifacts’ in fat histology (Figure 1B). Thus, this method was abandoned, considering also the successful staining obtained on paraffin.

In addition, relating quantitative analyses of FAT (±SVF), as previously published [17], from adipose tissue, by manual extraction, we obtained approximately 250,000 ± 34,782 nucleated cells per milliliter of fat tissue. Using the automatic extractor, however, cell yield was approximately 50,000 ± 6,956 nucleated cells per milliliter of fat tissue (*p* < 0.01).

### 3.3. Dissecting Fat Biopsies Areas by Histological Analyses

A careful microscopical analysis of H&E stained sections revealed high variability of the histological features, presumably linked to the areas of harvest into breast, to the quantity of harvested tissue and to the different timings between the harvest after the surgical treatments. Nevertheless, we were able to dissect the presence of 5 distinct fat histological features/areas. This distinction relies on our own experience in preclinical model of fat regeneration as well as on literature findings. 

**Figure 4:**
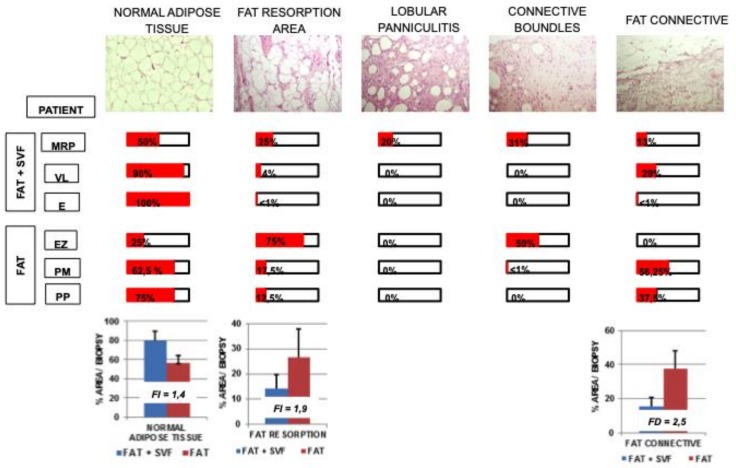
Tissue distribution of identified histological features within the sections for randomized selected patients.

Figure 4 summarizes tissue distribution of identified histological features within the sections for all patients. These considered histological features are:

- Normal Adipose Tissue. It is a physiological adipose tissue without any abnormalities.
- Areas Fat Resorption. This is anomalous adipose tissue composed by inflammatory cells infiltrating the lobules of adipocytes and associated by small/medium size cysts delimited by polimorphonucleated cells. On average, these adipocytes have a higher diameter compared to normal adipocitic cells.
- Lobular Panniculitis. This is an area of late stage fat resorption associated with diffuse amorphous stromal tissue containing numerous granulomatous syncytial-like structures and adipocytes-shape cysts. This area is also reported as necrosis. Thin connective bundles may also appear within the necrotic tissue.
- Connective Bundles. These are areas of amorphous and disorganized connective fat tissue, generally due to a further evolution of the necrotic event.
- Fat Connective. This is a physiological connective tissue associated with adipose tissue.

The prevalence of each area for section was quantified by visual scoring, as following reported:

+ scarcely displayed into the section
++ moderately displayed into the section
+++ significantly displayed into the section
++++ extensively displayed into the section

The score was assigned to any biopsy section and then the average score of all biopsies was calculated for each of the patients. The scorings were then converted into percentages as follows: + = 25%.

As reported in Figure 4, 2 out 3 samples of FAT + SVFs group (VL and E) displayed extensive presence of normal adipose tissue associated with scarce fat resorption and an absence of necrosis. Only one sample (MRP) displayed significant areas of necrosis and fat resorption, i.e., a percentage of about 20%. Curiously, MRP was a patient with a worst clinical history before the autologous fat transplant due to massive breast radiotherapy as adjuvant treatment which was associated with the formation of thick subcutaneous fibrotic layer. 

Similarly, 2 out 3 patients of FAT group (PM and PP) displayed significant levels of normal adipose tissue associated with moderate fat resorption and absence of necrosis. The third patient (EZ) revealed moderate levels of connective bundles without necrosis. 

Considering the normal adipose tissue as parameter, FAT + SVFs group showed an average of 79.17 ± 10.62% of this feature versus 56.25 ± 7.83% in FAT group, corresponding to a fold increase (FI) of 1.4 in normal adipose tissue. In the same line, it was possible to show an increase of fat resorption in the FAT only group, with an average of 14.35 ± 5.29% for FAT + SVF group versus 26.63 ± 11.13 for FAT group, corresponding to fold of increase of 1.9 in tissue resorption. Similarly, connective associated fat tissue was less present in FAT + SVFs group (15.28 ± 5.42%) versus FAT specimens (37.5 ± 10.56%), with a fold of decrease (FD) of 2.5 (Figure 4). The absence of statistical significance (by t-test) of these histological features between the groups is probably due to the low number of biopsies analyzed; thus, it will be interesting to evaluate the statistics of these analyses, possibly as new patients are recruited. It also seems valid for the other considered histological features that did not appear to be statistically different between FAT + SVFs versus FAT.

### 3.4. Assessment of Fibrosis in Breast Biopsies

**Figure 5:**
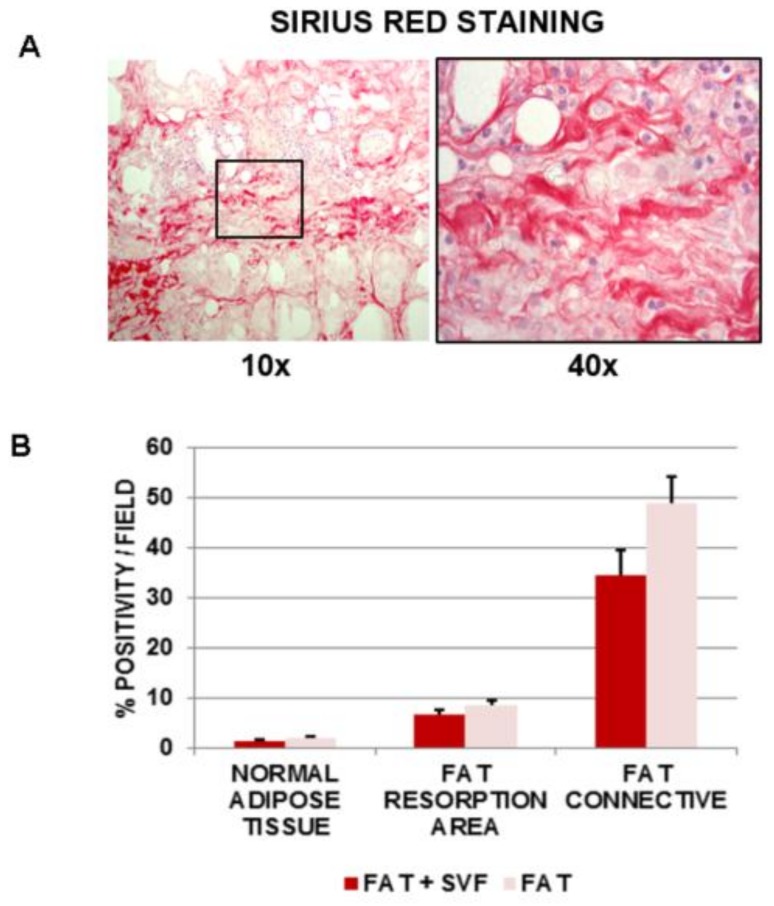
(**A**) Sirius red staining image to evaluate the grade of fibrosis in the FAT and FAT + SVF samples. 100× magnification (left) and 400× (right). (**B**) Sirius red quantification was performed for each of the distinct fat features/areas in the two groups.

Considering these findings, we then investigated the possible differences in fibrosis among FAT + SVFs versus FAT group. Sirius red staining was performed to evaluate the grade of fibrosis in the samples (Figure 5A). Fibrosis is attributed to excessive synthesis and deposition of extracellular component elements and subsequent interstitial deposition of collagen fibers as bundles. This is visualized by the intense red color of the Sirius red stain. Furthermore, it has been reported that the amount of fibrosis in fat depots could be associated with fat mass loss, typically after a process of reabsorption of adipose lobules. 

A shows a representative image of Sirius red stained adipose tissue (100× magnification and inset 400×) (Figure 5A) displaying the histological features of a lobular panniculitis, where the intense red color denotes the dense fibrotic connective bundles inside the necrosis, while the other granulomatous parenchymal tissue appeared pink in color. This latter feature (pink) has been excluded from the analyses. Thus, Sirius red quantification was performed for each of the distinct fat features/areas in the two groups. Date are reported in Figure 5B.

Within the normal adipose tissue, the level of Sirius red positivity resulted in lower than 2% both FAT + SVFs and FAT alone (1.28 ± 0.28% and 1.85 ± 0.25%, respectively). This parameter significantly increased in the region of fat resorption with a higher level in the FAT alone group. 

Taking into account fat connective tissue, Sirius red staining was highly predominant in the fat alone specimens, with more than 50% fibrotic tissue in this area. Lower levels of Sirius red stained fibrosis could be detected in the FAT + SVFs area. 

Although present in one specimen only (MRP from FAT + SVFs group), we also considered the fibrotic areas within lobular panniculitis. The analysis displayed a percentage of Sirius red positivity of 23.70 ± 4.94% (n = 10 HP fields analyzed) that is higher than the levels in normal adipose tissue and fat resorption area from FAT + SVFs specimens (not shown). Curiously, this sample was taken from woman with the worst clinical history before the autologous fat transplant, due to massive breast radiotherapy as adjuvant treatment being associated with the formation of thick subcutaneous fibrotic layer. Another interesting aspect linked to this latter patient was the investigation of fibrosis inside the connective bundles. Although this fat feature is present in this patient only (MRP) from FAT + SVF samples, and in two patients (PM and EZ) from the FAT group, our pilot analyses revealed surprisingly higher levels of fibrosis in FAT + SVF patient versus FAT patients (49.57 ± 3.97 versus 29.39 ± 3.91, respectively).

### 3.5. Angiogenesis in Breast Biopsies

After the analyses of fibrosis, we then proceeded to assess the angiogenesis in breast biopsies. In particular, we considered 2 distinct markers: CD31, endothelial marker for both mature and immature vessels, and l’α-SMA, as pericytic marker staining the extra endothelial part of mature vessels. 

**Figure 6:**
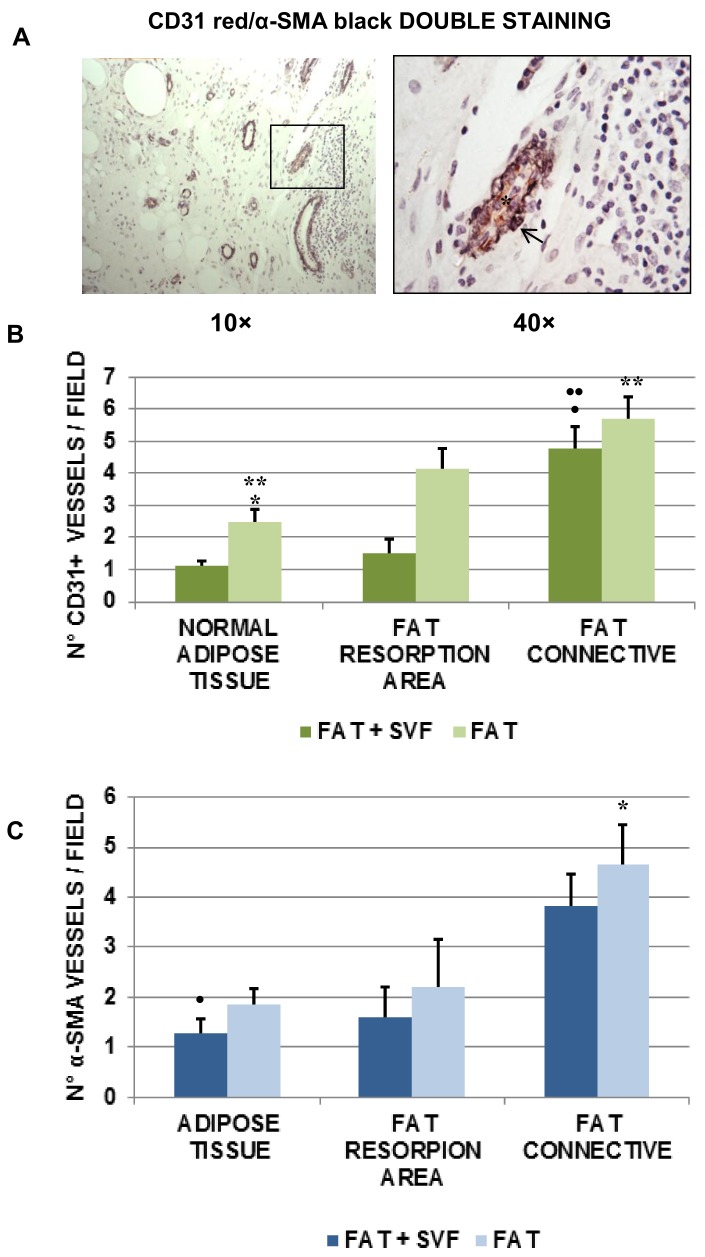
(**A**) Image of double staining red-CD31+ (star; novared as chromogen) and black-α-SMA+ (arrow; DAB-Nickel as chromogen) within a fat biopsy. The double stain shows a red color in the endothelial layer and a black color in the pericytic layer (4× inset). To evaluate the level of angiogenesis, both CD31 and α-SMA+ vessel density were considered. (**B**) The scoring for CD31+ vessel alone. (**C**) The scoring for α-SMA+ vessel alone. The symbols *, **, •, •• indicate the outliers.

Figure 6A displays a representative image of double staining red-CD31+ (star; novared as chromogen) and black-α-SMA+ (arrow; DAB-Nickel as chromogen) within a fat biopsy. The double stain shows a red color in the endothelial layer and a black color in the pericytic layer (Figure 6A, 4× inset). To evaluate the level of angiogenesis, both CD31 and α-SMA+ vessel density were considered. The scoring is reported in Figure 6B (for CD31+ vessel alone) and 4C (for α-SMA+ vessel alone).

**Figure 7:**
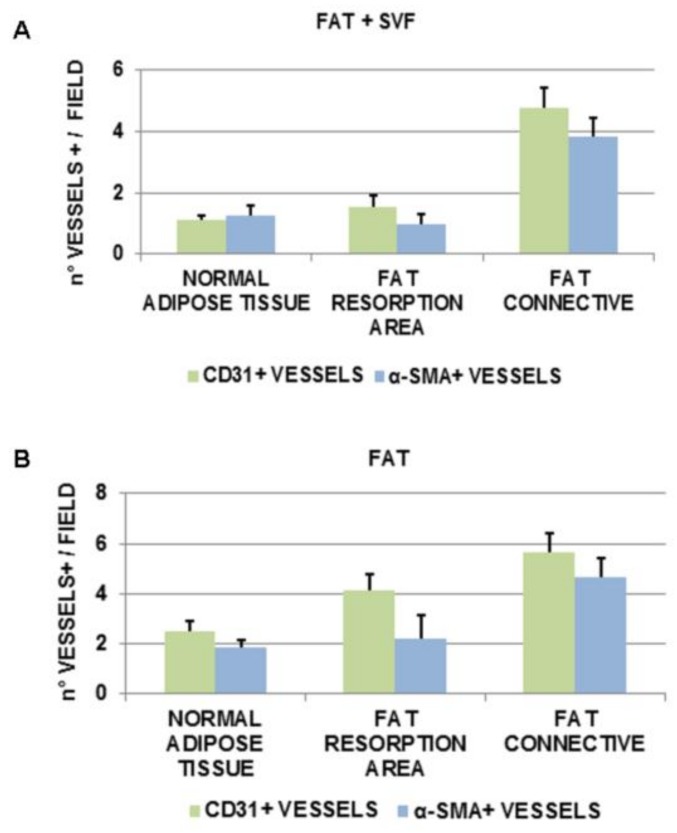
Angiogenesis data. (**A**) FAT + SVFs group showed similar amount of CD31+ and α-SMA+ vessels (*p* > 0.05), demonstrating the prevalent presence of mature vessels into the considered areas. This phenomenon was not totally reproducible in FAT group (**B**) that has higher, but non-significant, numbers of CD31+ vessels in comparison with to α-SMA+ vessels.

Collectively, both CD31+ and α-SMA+ for each of the analyzed fat features/areas were higher for FAT specimens versus FAT + SVF samples. In particular, comparing normal adipose tissue and the fat resorption area, the differences for CD31 stain among the two groups were statistically significant (# *p* = 0.002 and ## *p* = 0.003, respectively), while the same was not true for α-SMA (*p* > 0.05). Similarly, the higher amount of vessel density (CD31+ and α-SMA+) of connective associated fat tissue relative to FAT samples was again higher than FAT + SVF samples, but not statistically significant. Focusing on the anatomical features/areas inside each group, was could additionally observe that, comparing normal adipose tissue versus fat resorption areas, the FAT + SVF samples displayed similar amount of CD31+ and α-SMA+ vessels (*p* > 0.05/each). Instead FAT samples, displayed different amount of CD31+ vessels (increased count for fat resorption feature/area; * *p* = 0.03), but similar amount of α-SMA+ vessels. Comparing normal fat tissue to connective associated fat tissue inside the same group, both the FAT + SVF and FAT groups displayed a significative higher amount of vessels (CD31+ and of α-SMA+) in the connective fat tissue (*p* < 0.05). Furthermore, connective associated fat tissue displayed more vessels (CD31+ and α-SMA+) compared to the fat resorption area for both groups; however, the difference was significant only for CD31+ cells and exclusively for the FAT + SVFs group. To better visualize the findings referred to the observed angiogenesis, the data have been also organized for patient groups (Figure 7). Considering the FAT + SVF group (Figure 7A), there were similar amounts of CD31+ and α-SMA+ vessels (*p* > 0.05), demonstrating the prevalent presence of mature vessels into the considered areas. This phenomenon was not totally reproducible into FAT group (Figure 7B) that have higher, but non-significant, numbers of CD31+ vessels in comparison to α-SMA+ vessels.

### 3.6. Expression of Embryonic and Neurogenic Stem Cells Markers

**Figure 8:**
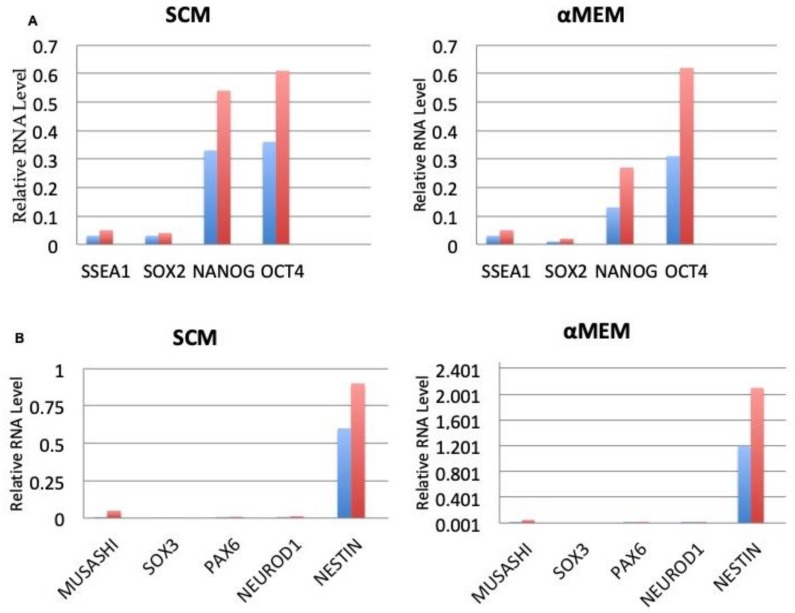
Pluripotency associated endogenous gene expression. (**A**) Expression of SOX2, NANOG, OCT4, SSEA1and (**B**) Expression of NESTIN, NEUROD1, PAX6, SOX3 and MUSASHI in FAT + SVF sample maintained in αMEM and SCM was investigated by quantitative Real Time RTPCR. The assay was performed in triplicate in 4 different cases with similar results. The quantitative expression of genes of interest relative to the housekeeping gene HPRT1 (blue columns) and GUSB (red columns) was calculated. Data are expressed as the mean of four independent experiments ± SD. Analysis of results for statistical significance was performed with the student’s T-test.

RT-PCR was used during this study to examine the molecular properties and plasticity of self renewal. After testing, it was found that hASCs experienced growth in both media, expressed in a spontaneous fashion at the same type of level of embryonic stem cell genes, including SSEA1, OCT4, NANOG, and SOX2 (Figure 8A). Furthermore, the growth was positive in terms of the expression of genes linked with the neurogenic lineage, as found through the gene profile. This would include NEUROD1, PAX6, NESTIN, SOX3, and MUSASHI (Figure 8B). Specifically, the neurogenic characterization of gene expression demonstrated that the αMEM culture had positive results in terms of PAX6, whereas the opposite was true for SOX3. In terms of the last two genes, the SCM cultures exhibited the opposite expression profile. 

## 4. Discussion

Mesenchymal stem cells that are derived from adipose tissue, alongside fat, make up a major field of research in regenerative plastic surgery. The easy access to fat, through minimum invasive techniques such as liposuction, makes it a primary and easy source of hASCs [19]. This study has found that hASCs that express self-renewal and possess multi-lineage potential can be acquired through the enzymatic digestion of fat graft. The acquired hASCs have been examined using different methods, including real time RT-PCR, flow cytometry, and immunocytochemistry. The hASCs were split from adipose tissue taken from 23 patients, and reliably fulfill the general definition of MSCs by both phenotypic and differentiation capabilities criteria [20].

In the absence of enzymatic digestion of fat tissue, the chance of clinical translation of the multi-lineage potential of these cells is delayed by the poor/negligible cell survival within cryopreserved lipoaspirates, the difficulty of ex vivo expansion, and the complexity of current Good Manufacturing Practice (cGMP) requirements for expanded cells [21]. Hence, availability of a minimally manipulated, autologous, SVFs-enriched fat product would have remarkable clinical relevance. Bianchi et al. [21] presented a Lipogems system, providing a non-expanded, ready-to-use fat product. The system uses mild mechanical forces in a completely closed system, avoiding enzymes, additives, and other manipulations. Immunohistochemistry revealed that Lipogems stromal vascular tissue included abundant cells with pericyte/hMSC identity [21]. Flow cytometry analysis of a non-expanded, collagenase-treated Lipogems product showed that it was comprised with a significantly higher percentage of mature pericytes and hMSCs, and a lower amount of hematopoietic elements than enzymatically digested lipoaspirates. In a study by Shah et al. [22], the authors evaluated the isolation of hASCs without enzymatic digestion. They used a simple method, i.e., washing adipose tissue, to isolate and characterize the cells and compared this procedure with the enzymatic digestion in terms of processing time, stem cell yield, differentiation potential and immunophenotype. Based on fluorescence-activated cell sorting analysis, the SVFs isolated with the washing method displayed a distinct and potentially favorable immunophenotype relative to the collagenase digestion. This difference may reflect the absence of chemical alteration of the cells by enzymatic digestion. Independent of the isolation procedure, the resulting passaged hASCs were comparable, based on immunophenotype and adipogenic and osteogenic differentiation potential. Although using collagenase substantially increases cell yield, the two methods yield a similar cell product. For these reasons, collagenase could be considered an expensive reagent derived from a bacterial source, and its use in isolating hASCs could be considered time-consuming.

The authors support the use of SVF-enhanced fat grafts obtained by enzymatic, and not enzymatic, digestion (both), but feel the necessity to report, in the present study, the differences in term of angiogenis and fibrosis.

hASCs can be recruited under certain stimuli and committed to become preadipocytes, and then mature adipocytes. Controlling stem cell differentiation towards the adipogenic phenotype could have a great impact on future drug development aimed at counteracting fat depots [23]. As reported in a study of Basoli et al. [23], ASCs commitment can be influenced by melatonin, which the authors showed to be an osteogenic inducer. The transcription of specific adipogenesis orchestrating genes, such as aP2, peroxisome proliferator-activated receptor γ (PPAR-γ), and that of adipocyte-specific genes, including lipoprotein lipase (LPL) and acyl-CoA thioesterase 2 (ACOT2), were significantly inhibited in cells that had been treated in the presence of melatonin [23]. Protein content and lipid accumulation confirmed a reduction in adipogenesis in ASCs that had been grown in adipogenic conditions, albeit in the presence of melatonin and/or vitamin D [23].

In addition, preclinical experiments conducted by Tian et al. [24] have demonstrated that transplanted ASCs can improve neurological functions, reduce small regions of cerebral infarction, promote angiogenesis, and express neuron-specific markers. The improvement of neurological functions was demonstrated in experiments using different methods and time courses of ASCs transplantation, although the mechanisms remain unclear [24].

Furthermore, these ASCs exhibit an increased level of neural marker expression [25], which is associated with the capacity to produce neuropheres. The procedure to acquire hASCs is fairly sluggish, albeit safe. It does not need any digestion of enzymes with collagenase [9,26]. Through the direct enzymatic digestion during culture of the adipose tissue, it becomes likely that a large number of cells can be acquired. This was possibly the main element that caused the tissue source to experience fine intrinsic changes. The morphological evaluation of the SVF-enhanced fat tissue showed a stromal structure, which is significantly preserved and does not lose its constitutive elements at the same quantitative distribution or density. The structural definition at play also remained consistent post digested-tissue cryopreservation.

Immunohistochemical analyses showed that hASCs expressed classical mesenchymal markers such as CD44, CD73, CD90, CD105 and CD166 [20,27]. The endothelial (CD31, CD34, CD144, CD146) and hematopoietic (CD45, CD133) markers were much less represented, and (as expected) the expression of some of these markers was modified at higher number of passages [28]. In particular, a time-dependent decrease of CD133/2 marker was observed in SVF-enhanced hASC grown in SCM medium/negative CD133 (data not shown). Such human neural stem cells exhibited higher efficiency in colony formation than CD133 positive cells [29]. This effect could be related to the likelihood that CD133 expression appears linked to cell cycle phase in neural stem cells [30]. It would be interesting to investigate whether CD133 expression might be related to the cell cycle phase in the case of our isolated cells. The CD105 increase in both media, or the opposite situation for CD144 and CD146 in the two media, has been also reported recently by other authors, which showed that these variations can be ascribed to the presence of supplements such as growth factors (FGF-2), cytokine (IL-6), ascorbic acid, or transferrin in the culture medium [28]. A hallmark of MSCs is their multi-potency and ability to give rise to tissue of mesenchymal origins, such as osteoblastic, chondrogenic and adipogenic lineages. These features were present in hASCs obtained from digested product, and were also preserved at higher passages, although the in vitro test of fat accumulation demonstrated qualitative and quantitative differences between digested fat derived cells and those obtained from lipoaspirate. 

This observation again indicated that sub-cellular changes were triggered by the digestion of the adipose tissue with reduced ability to form fat and a higher one in the ability of forming bones precursors. 

This indication is further supported by the higher propensity towards neural phenotype of these cells, compared to classical PLA- derived hASCs. The aggregation of MSC into 3D spheroid may increase their ability to differentiate and enhance their potential therapeutic properties [31]. In contrast, Carelli et al. [25] found that only hASCs obtained by fragmented fat tissue (not digested tissue) efficiently formed spheroids when grown in neurosphere-forming medium (in absence of mitogenic stimuli), and expressed molecular markers typical of neuronal lineage at very high levels, when grown in basal growth conditions. In addition, EPOR was expressed when these cells were grown in SCM. These findings provide further evidence that digestion and fragmentation of lipoaspirate adipose tissue changed some molecular features in the tissue resident cells so that hASCs obtained in this condition became more plastic and adaptive to the extracellular signals. Approaches aimed at understanding the molecular changes triggered by the digestion are now in full development, and will be subject to a near future publication. Nanog, Oct-4 and Sox-2 are three transcription factors expressed at high levels in embryonic stem cells (ESCs). They regulate the expression of other genes during development and are found at high levels in the pluripotent cells of the inner cell mass [25]. The down-regulation of these three transcription factors correlates with the loss of pluripotency and self-renewal, as well as the beginning of subsequent differentiation steps [32]. These genes are expressed in some mesenchymal stem cells such as bone marrow stem cells [33], term amniotic fluid stem cells [34], and breast milk stem cells [35]. The gene profile assays of the current study found that SVFs-enhanced hASCs, grown either in αMEM or SCM culture media, expressed OCT4, OCT4- dependent SOX2 and NANOG that are transcription factors responsible for embryonic stem cell self-renewal with pluripotency [36]. Through gene expression profile, this study demonstrated that these cells express neurogenic lineage factors, such as NESTIN, NEUROD1, MUSASHI1, PAX6 and SOX3). Also, the immune-fluorescence staining indicated a higher expression of neural stem markers in hASCs which consequently co-expressed nestin, β-tubulin III, glial GFAP and O4 [25]. Notably, the above phenotypic profile was reported at lower extent by other authors in mesenchymal stem cells obtained from different sources, such as bone marrow [37] amniotic fluid [38], nucleus polposus [39] and adipose tissue [40]. The ability of undifferentiated MSC to express immature and mature proteins typical of other tissues without any induction may support their plasticity to differentiate easily in many various tissues [41]. These features suggest that the pluripotency nature of hASCs cells, and highlight their novelty for regenerative studies and those focused on molecular determinants of stem cells.

The potential benefit of SVFs pellets and SVF-enhanced fat graft in tissue regeneration could be explained by the ability of stromal cells, to secrete various growth factors that improve angiogenesis [42], leading to an increased survival of vessels, as documented in this study. The authors feel the necessity to report with this study that the mechanism of tissue regeneration could be represented by (1) targeting of damaged zone, (2) angiogenic and antiapoptotic factors release followed by formation of new vessels and oxygenation. Angiogenesis was showed in this study and confirmed from histological analysis highlighting there were similar amount of CD31+ and α-SMA+ vessels (*p* > 0.05), demonstrating the prevalent presence of mature vessels (α-SMA+ vessels) into the considered areas treated with SVF-enhanced fat graft (FAT + SVF). This phenomenon was not totally reproducible into FAT group that have a higher, but non-significant, number of CD31+ vessels (immature vessels) in comparison with to α-SMA+ vessels.

SVFs-enhanced fat graft improved angiogenesis and fibrogenic activity of fibroblasts [17]. The results reported offer an ex vivo analysis and in vivo approach of fat graft, that makes it clear how one is fundamental provides an optimized micro-environment, in tissue engineering, supporting the correct architectural adipocyte distribution and better cell-to-cell interaction. This concept could offer early protection from surrounding inflammatory events. The early establishment of new vessels and the proper deliver of nutrients and oxygen to the implant may contribute to the improved outcomes observed.

## 5. Conclusions

In conclusion, the use of the Celution™ apparatus to aid in the digestion of adipose tissue has made it possible for many beneficial results and discoveries relating to the tissue properties and the MSCs that have been derived as a result. This process did not alter the stomal structures and cell contents. These results indicate the potential usefulness of SVF-enhanced fat tissue and SVFs pellets in regenerative medicine, as opposed to the traditional lipoaspirate. The potential for therapeutic applications in personalized medicine based on autologous resource was shown by these cells, in outcomes of breast reconstruction.
